# Perioperative management of acute myocardial infarction in the 31st week of pregnancy: A case report and literature review

**DOI:** 10.3389/fsurg.2022.1023551

**Published:** 2022-11-02

**Authors:** Pei Sun, Xin-Quan Liang, Tao Hong, Hong Zhang

**Affiliations:** ^1^ Department of Anesthesiology and Critical Care Medicine, Peking University First Hospital, Beijing, China; ^2^ Department of Cardiology, Peking University First Hospital, Beijing, China

**Keywords:** pregnancy-related acute myocardial infarction (PAMI), anesthesia management, case report, pregnancy, multidisciplinary collaboration

## Abstract

Pregnancy-related acute myocardial infarction (PAMI) is rare but life-threatening. The incidence of PAMI is growing over time for multiple reasons, and the management of parturients with acute myocardial infarction is challenging in terms of diagnosis and treatment. To date, there are still no clear guidelines on the best practice for PAMI. We present a case of a 41-year-old woman with PAMI at 31 weeks of pregnancy. Through multidisciplinary collaboration, successful outcomes were achieved for both the mother and fetus.

## Introduction

Pregnancy-related acute myocardial infarction (PAMI) is relatively infrequent but is associated with notable maternal or fetal morbidity and mortality (1). The incidence of PAMI ranges from 0.06 to 10 per 100,000 pregnant women globally (2), with a case-fatality rate of 4.3% to 37% (3–5). The rate of PAMI is growing over time due to multiple reasons, such as rising maternal age and increased prevalence of obesity and diabetes (6). Spontaneous coronary artery dissection (SCAD) is a main etiology of PAMI and has been reported to occur in 15% to 40% of acute coronary syndrome (ACS) cases during pregnancy (2). To date, there are still many gaps in the understanding of PAMI, as guidelines for the management of PAMI are still limited (7). Management of puerperae suffering acute myocardial infarction could present significant clinical challenges to perinatal multidisciplinary teams (1, 8). We describe a case of a 41-year-old woman with PAMI at 31 weeks of pregnancy. Written informed consent was received prior to publication of this report.

## Case report

**Figure 1:**
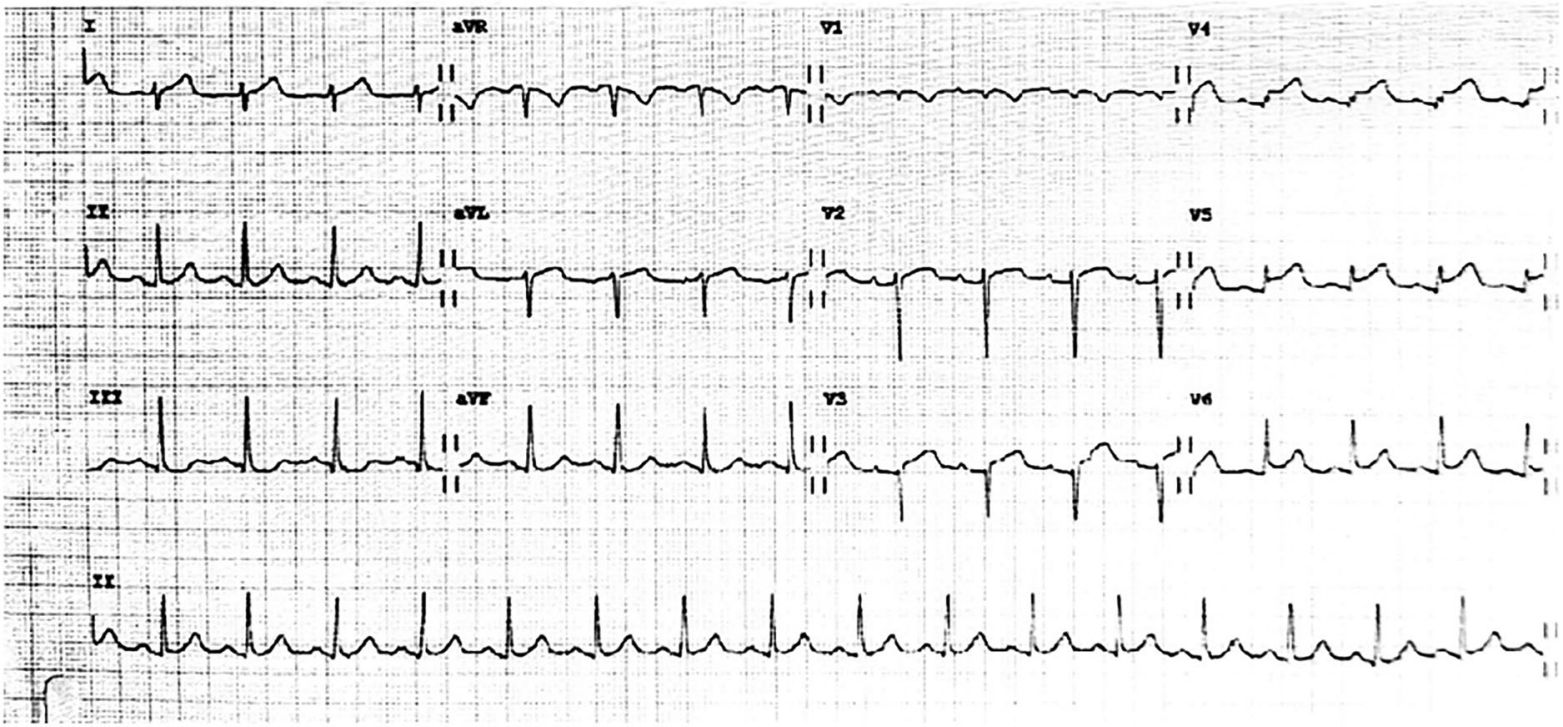
Twelve-lead electrocardiogram showed ST-segment elevation in leads I, aVL, and V2-V6.

A 41-year-old woman at 31 weeks gestation, gesta3, para1, with a history of previous correction surgery of patent ductus arteriosus, was admitted to the emergency department (ED) after suffering sudden crushing pain in the precordial area, accompanied by radiation on the left shoulder and back, toothache for 4 h. Electrocardiogram (ECG) showed ST-segment elevation in leads I, aVL, and V2–V6 (Figure 1) and the myocardial enzymes increased. The echocardiography demonstrated segmental wall dyskinesia (apex, interventricular septum) and the left ventricular ejection fraction (LVEF) was estimated to be 59.5%. In view of the patient's symptoms, signs and test results, the patient was diagnosed with ST-segment elevation myocardial infarction (STEMI). Epidemiological investigation of the patient on admission revealed that she had no history of COVID-19 infection and was not vaccinated due to pregnancy. The patient had no previous history of angina pectoris and no risk factors, such as hypertension, diabetes, smoking, alcohol consumption or family history of coronary heart disease. Therefore, spontaneous coronary dissection was highly suspected.

**Figure 2:**
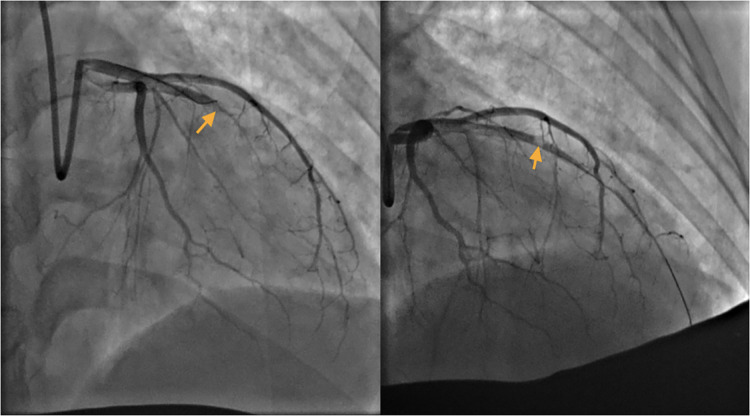
Left: Left anterior descending (LAD) artery: critical lesion in mid LAD. Right: The guide wire passed through the occlusion.

The patient's symptoms were not relieved, so an interventional cardiologist performed emergent percutaneous coronary intervention (PCI) for the puerpera. Cardiac angiography showed that the left anterior descending (LAD) artery was 100% occluded (Figure 2), and the right coronary artery showed no obvious abnormalities. After the guiding wire passed through the occlusion, the blood flow of the anterior descending artery was opened (Figure 2). Stent placement was recommended for the patient due to unstable status, but the placement was unsuccessful owing to complex lesions.

A multidisciplinary team including an obstetrician, cardiologist, anesthesiologist, pediatrician and pharmacologist was established. The MDT considered pregnancy before 34 weeks has a high risk of cardiovascular events and low fetal survival and eventually reached a consensus that cesarean section would be performed two weeks after PAMI.

**Figure 3:**
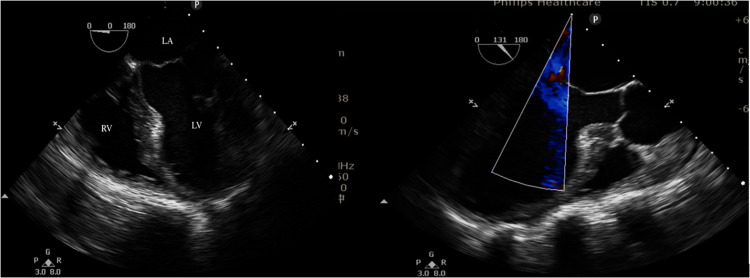
Intraoperative transesophageal echocardiograms.

Cesarean section was scheduled at 34 weeks and 3 days of gestation. After the patient entered the operating room, electrocardiography, pulse oxygen saturation, invasive blood pressure and bispectral index (BIS® Sensor; Aspect Medical Systems, Natick, MA, USA) monitoring were routinely monitored. Endotracheal intubation was performed after induction. Anesthesia was maintained using sevoflurane and remifentanil. Intraoperative temperature monitoring was performed with a temperature probe. Forced air warming and warmed intravenous fluids were used to maintain the patient's core temperature. A transesophageal echocardiographic probe was then inserted, and cardiac function was monitored in real time. The examination showed decreased left ventricular function. There were multiple wall motion abnormalities, including apical septal dyskinesia (Figure 3). No valvular abnormalities were noted. At the same time, the FloTrac/Vigileo System (Edwards Lifesciences, Irvine, CA) was applied for hemodynamic monitoring. Five minutes after intubation, a 2,300 g female baby was born. The Apgar scores for the neonates were 8 and 10 at 1 min and 5 min, respectively. No respiratory depression was observed in the neonate, and she was transferred to the NICU. We administered 10 µg of sufentanil to the woman after the baby was delivered. The total volume of crystalloid fluids administered was 600 ml, and blood loss at the end of cesarean delivery was approximately 400 ml. Ultrasound-guided transversus abdominis plane block was performed after cesarean section. The patient was safely extubated and then admitted to the cardiac care unit (CCU) with a sufentanil patient-controlled analgesia pump.

She was monitored for 6 days postoperatively in the CCU, without postpartum hemorrhage, anginal symptoms or ECG changes. Echocardiographic examination did not show significant alterations, and myocardial enzymes showed no evidence of new ischemia.

Ten days after the cesarean section, the patient was discharged from the hospital. The patient and the child performed well at 6-month and one-year follow-up.

## Discussion

PAMI is different from AMI in general patients in terms of etiology, diagnosis, and therapy. The management of pregnant patients with acute myocardial infarction is challenging and requires close cooperation between the obstetrician, cardiologist, neonatologists and anesthesiologist. Therefore, we need to better understand the pathophysiology and management of PAMI and formulate an individualized anesthesia protocol for maternal and fetal safety.

### The etiology and risk factors

Cardiovascular disease during pregnancy is the most common cause of pregnancy-associated mortality (9). AMI can occur throughout pregnancy but is more likely in the third trimester. PAMI is a multifactorial disease caused by multiple predisposing factors, such as hypertensive syndromes, known coronary artery disease (CAD), hyperlipidemia, thrombophilia states, substance abuse history, smoking history, obesity, multiple comorbidities, medicaid insurance status, and black race (6). Age >35 years is one of the most consistently reported risk factors for PAMI (10). The etiology of CAD in pregnancy differs from that in the general population; most CAD has nonatherosclerotic mechanisms, including pregnancy-related spontaneous coronary artery dissection (*P*-SCAD) (43%), angiographically normal coronary arteries (18%) and coronary thrombosis (17%) (6).

### Multidisciplinary collaboration

Pregnant women with cardiac disease require appropriate anesthesia, cardiac, and obstetric management during the perinatal and postpartum period. Guidelines from the American Heart Association (AHA), European Society of Cardiology (ESC), Society of Maternal Fetal Medicine (SMFM) and American College of Obstetricians and Gynecologists (ACOG) suggest a “Pregnancy Heart Team” care for pregnant patients with complicated cardiac disease (11). In our case, a multidisciplinary team including an obstetrician, cardiologist, anesthesiologist, pediatrician and pharmacologist was established to assess the severity of cardiovascular disease, develop treatment and delivery planning.

### Percutaneous coronary intervention

Myocardial revascularization and the rapid establishment of optimal cardiac treatment to limit cardiac remodeling are emergency interventions to improve maternal cardiac prognosis. The goal of management in the acute phase is to restore or preserve myocardial perfusion and cardiac function. The majority of AMI during pregnancy is caused by spontaneous coronary hematoma/dissection. The mechanism is unknown and may be related to changes in hormone levels during pregnancy. The mechanism of atherosclerosis is completely different from that of common AMI patients. In principle, if coronary angiography suggests a subintimal hematoma, as long as the vessel is not completely occluded and forward blood flow exists, then PCI is not recommended. Otherwise, the subintimal hematoma will extend and cause more problems. Generally, the hematoma is completely absorbed, and the blood vessel returns to normal in approximately one month. However, it was impossible to distinguish whether it was a hematoma or a thrombosis secondary to common plaque rupture during angiography in this patient; moreover, the blood vessel was completely occluded, so interventional treatment had to be performed. It was very difficult to pass the guiding wire, and guiding wires with different hardnesses were replaced three times. Finally, the occlusion segment was forcibly passed with the assistance of the microcatheter. Angiography showed no calcification in the local area. Any new devices, including balloons, stents, and intravascular ultrasound catheters, could no longer pass through the occlusion, and the cardiologist determined that it was caused by endothelium prolapse, which blocked the lumen. On the other hand, the biggest concern of PCI in pregnancy is radiation exposure. According to previous research, the radiation exposure of patients during procedures such as coronary angiography is estimated to be <20 mGy, while the fetal share of radiation is estimated to be 0.074 mGy. At doses below 50 mGy, there is a negligible risk of fetal teratogenicity following radiation exposure, while fetal damage may occur at doses above 150 mGy (2). Therefore, we believe that the radiation dose in coronary angiography is safe for most pregnant patients.

### Antiplatelet therapy

Research on antiplatelet drugs is limited because pregnant and breastfeeding women are often excluded from these studies. Low-dose aspirin appears to be safe and can be continued during delivery (1, 9, 10). For patients whose main mechanism is spontaneous subintimal hematoma, it is generally not recommended to take antiplatelet and anticoagulant drugs (and statins) to avoid aggravating subintimal hemorrhage. However, in this case, the patient had undergone PCI because of endothelium prolapse and subsequent blocking of the lumen. The blood flow was restored by PCI with severe residual stenosis, and the stent was not successfully implanted. Therefore, antiplatelet drugs were administered to prevent the reocclusion of blood vessels caused by local thrombosis.

### Anticoagulation therapy

Anticoagulation with heparin in pregnant patients is considered safe, as it does not cross the placenta and therefore has no teratogenic effect. Low molecular weight heparin (LMWH) is generally the preferred agent due to its easier administration and more predictable effect. Neuraxial anesthesia should be avoided for at least 12 h after subcutaneous prophylactic dose of LMWH and at least 24 h for therapeutic dose of LMWH according to The American Society of Regional Anesthesia (ASRA) Guidelines and the SOAP Consensus Statement (12, 13).

### Timing of delivery

For women with acute myocardial infarction during pregnancy, choosing the right timing for delivery is crucial. In the case of acute myocardial infarction, delivery should be postponed a few weeks after AMI to reduce hemodynamic stress immediately after the event (9), if possible, for at least two weeks from the time of infarction. In previous relevant case reports, most patients underwent elective cesarean section or vaginal birth after two weeks (Table 1). The risk of myocardial infarction complications was high within 2 weeks after AMI, and the fetal lung was immature. Considering the improved cardiac function after two weeks of medical treatment, a multidisciplinary team decided to postpone cesarean section until 34 gestational weeks.

**Table 1:** Summary of case reports of anesthesia management for acute myocardial infarction (AMI) in pregnancy.

Ref.	Type	Maternal age	PAMI time	LVEF	Timing of delivery	Mode of Delivery	Anesthesia tenique
Hands et al (14), 1990	Case report	26	38 weeks	UK	40 weeks	CS	Epidural anesthesia
		31	36 weeks	48%	64 h after AMI	CS	Epidural anesthesia
Gil et al (15), 2006	Case report	31	38 weeks	57%	38 weeks	CS	General anesthesia
Duarte et al (16), 2011	Case report	39	31 weeks	30%	35 weeks	CS	Epidural anesthesia
Pougeoise et al (17), 2012	Case report	32	38 weeks	45%	25 h after AMI	CS	General anesthesia
Frassanito et al (18), 2012	Case report	36	25 weeks	57%	35 weeks	CS	General anesthesia
Mihaljevic et al (19), 2015	Case report	32	20 weeks	41%	38 weeks	CS	General anesthesia
		35	30 weeks	68%	38 weeks	Vaginal birth	Epidural anesthesia

PAMI, pregnancy-related acute myocardial infarction, AMI, acute myocardial infarction, LVEF, left ventricular ejection fraction, UK unknown, CS, cesarean section.

### Mode of delivery: Vaginal or cesarean delivery?

There is no general consensus on the recommended mode of delivery for pregnant women with AMI. The advantages of vaginal delivery include less blood loss and a lower risk of infection, venous thrombosis, and embolism and should be recommended for most women (9). Mihaljevic et al. (19) reported two cases of women with PAMI, one of whom was submitted to vaginal delivery due to her good clinical status. Cesarean section (CS) appears to be the most stable and controllable delivery method for parturients suffering from an acute cardiac event because it allows better control of labor time and prevents stress responses due to prolonged vaginal childbirth. The rate of CS in pregnancy-onset ischemic heart disease (IHD) is 62%–84.6% (20, 21). Elective CS generally does not improve maternal or fetal outcomes in women with stable heart disease. Baris et al. (20) considered that elective CS should be mainly recommended for high-risk obstetric indications, such as after a recent AMI or if the left ventricular ejection fraction continues to decline. However, the risk of having an adverse cardiovascular event was significantly greater in the cesarean group than in the vaginal delivery group (22), and the multidisciplinary team needs to pay more attention to maternal recovery after surgery. Roth et al. (23) in their review, held the opinion that the mode of delivery in patients with PAMI should be determined by obstetric indications and the clinical status of the mother. An appropriate and individualized decision based on the clinical status of the patient is the key for the optimal mode of delivery.

### Anesthetic technique: Regional anesthesia or general anesthesia?

The decision to choose regional or general anesthesia is multifaceted. Both regional and general anesthesia have advantages and disadvantages in pregnant women with acute myocardial infarction. It is preferred to choose neuraxial anesthesia for cesarean delivery whenever possible in women with cardiac disease (11). In Yildırım's study (24), the proportion of regional and general anesthesia used during cesarean section was similar among parturients with heart disease, but it was observed that general anesthesia was mainly preferred for parturients with higher NYHA classifications, requiring emergency surgery, history of previous cardiac surgery or medication, and stage 3 or higher multiple valvular disease. Gil et al. (15) reported a case of PAMI presenting for CS under general anesthesia. They used rapid sequence induction with rocuronium and etomidate. The patient was monitored with direct blood pressure and central venous pressure and did not present clinical signs of hemodynamic instability during the course of the surgery. Considering that the woman was in the acute phase of myocardial infarction and was being treated with oral anticoagulants, the MDT decided to perform a cesarean section under general anesthesia.

### Intraoperative anesthesia monitoring techniques

Intraoperative anesthesia management goals include ensuring coronary perfusion and avoiding tachycardia and excessive ventricular end-diastolic volume, maintaining cardiac output and myocardial contractility, adequate arterial oxygen content, and maintaining body temperature and internal environment stability.

Adequate hemodynamic monitoring is essential to reduce perioperative morbidity and mortality in cardiac patients. On the one hand, intraoperative transesophageal echocardiography (TEE) can be used as an important tool to dynamically monitor the changes in cardiac function of patients with severe comorbidities or if hemodynamic instability is expected or occurs intraoperatively. In the absence of ECG changes during myocardial ischemia, regional wall motion abnormalities have been reported in a significant proportion of patients. Because mechanical abnormalities (systole and diastole) precede electrical abnormalities during ischemia, TEE has the advantage of early identification of myocardial ischemia (25). On the other hand, the FloTrac/Vigileo system provides important information on hemodynamic status, such as cardiac output (CO), cardiac index (CI), stroke volume (SV), and stroke volume variation (SVV) (26) and it provides a useful method to determine the differential diagnosis of circulatory failure, especially to distinguish among cardiac factors, vascular factors, and blood volume. Consequently, it provides a method to monitor hemodynamic status, changes in the clinical course and responses to therapeutic interventions in patients who have arterial catheters in place. This technique may be potentially useful and suitable for high-risk pregnant women to guide therapy with fluids and vasoactive drugs. As far as we know, this is the first case to describe the application of FloTrac/Vigileo and TEE for cesarean delivery.

### Postoperative analgesia

The postpartum period is a time of increased risk of cardiovascular disease-related maternal morbidity and mortality (27). Postoperative pain is an important risk factor that contributes to postoperative myocardial ischemia and MI (28). Cesarean section can cause moderate to severe acute postoperative pain. Improper postoperative pain control may delay the mother's recovery, interfere with breastfeeding, and have a negative influence on mother-infant bonding (29). Therefore, it is important for anesthesiologists to seek optimal postoperative analgesia for parturients with cardiac disease. At present, multimodal analgesic strategies, including neuraxial anesthesia, peripheral nerve blocks, and administration of nonopioid analgesics, are widely performed after cesarean section. In the past, it was reported in the literature (16, 26) that neuraxial anesthesia for women with acute myocardial infarction who underwent cesarean section after stopping antiplatelet drugs before surgery has been shown to provide safe and superior postoperative analgesia and keep hemodynamic parameters more stable. For parturients undergoing general anesthesia, postoperative patient-controlled intravenous analgesia with sufentanil (PCA) plus TAP block is an alternative to neuraxial analgesia. USG-TAP block provides effective analgesia in women receiving CS, helps improve the severity of nausea and vomiting, and has good maternal satisfaction (30). During the postoperative follow-up, the patient was satisfied with the analgesic effect.

## Conclusion

The management of parturients with acute myocardial infarction is challenging in terms of diagnosis and treatment, and successful outcomes for both the mother and fetus come from the efforts of a multidisciplinary team of obstetricians, cardiologists, anesthetists and pediatricians. There are still no clear guidelines on the best practice for the PAMI. Our case demonstrates that multidisciplinary collaborative management, precise timing of surgery, and individualized perioperative management may help improve maternal outcomes and neonatal health for pregnant women with PAMI undergoing cesarean delivery.

## Data Availability

The raw data supporting the conclusions of this article will be made available by the authors, without undue reservation.

## References

[1] Alameh A Jabri A Aleyadeh W Nasser F Al Abdouh A Kondapaneni M Pregnancy-associated myocardial infarction: a review of current practices and guidelines. Curr Cardiol Rep. (2021) 23(10):142. 10.1007/s11886-021-01579-z 34410528

[2] Merlo AC Rosa GM Porto I. Pregnancy-related acute myocardial infarction: a review of the recent literature. Clin Res Cardiol. (2022) 111(7):723–31. 10.1007/s00392-021-01937-5 34510263 PMC9242969

[3] Gibson P Narous M Firoz T Chou D Barreix M Say L Incidence of myocardial infarction in pregnancy: a systematic review and meta-analysis of population-based studies. Eur Heart J Qual Care Clin Outcomes. (2017) 3(3):198–207. 10.1093/ehjqcco/qcw060 28838086 PMC5862024

[4] Hankins GD Wendel GD Jr. Leveno KJ Stoneham J. Myocardial infarction during pregnancy: a review. Obstet Gynecol. (1985) 65(1):139–46. PMID: 3966016

[5] Tripathi B Kumar V Pitiliya A Arora S Sharma P Shah M Trends in incidence and outcomes of pregnancy-related acute myocardial infarction (from a nationwide inpatient sample database). Am J Cardiol. (2019) 123(8):1220–7. 10.1016/j.amjcard.2019.01.030 30803707

[6] Balgobin CA Zhang X Lima FV Avila C Parikh PB Yang J Risk factors and timing of acute myocardial infarction associated with pregnancy: insights from the national inpatient sample. J Am Heart Assoc. (2020) 9(21):e016623. 10.1161/JAHA.120.016623 33106090 PMC7763409

[7] Prudhvi K Jonnadula J Rokkam VRP Kutti Sridharan G. Pregnancy associated spontaneous coronary artery dissection: a case report and review of literature. World J Cardiol. (2021) 13(4):103–10. 10.4330/wjc.v13.i4.103 33968309 PMC8069519

[8] Ng KX Li KF Tan CK Ong PJ. Non-ST elevation myocardial infarction in pregnancy-a critical review of current evidence and guidelines. Rev Cardiovasc Med. (2021) 22(4):1535–9. 10.31083/j.rcm2204157 34957792

[9] Regitz-Zagrosek V Roos-Hesselink JW Bauersachs J Blomstrom-Lundqvist C Cifkova R De Bonis M 2018 ESC guidelines for the management of cardiovascular diseases during pregnancy. Eur Heart J. (2018) 39(34):3165–241. 10.1093/eurheartj/ehy340 30165544

[10] Tweet MS Lewey J Smilowitz NR Rose CH Best PJM. Pregnancy-associated myocardial infarction: prevalence, causes, and interventional management. Circ Cardiovasc Interv. (2020) 13(11):e008687. 10.1161/CIRCINTERVENTIONS.120.008687 PMC7854968 32862672

[11] Meng ML Arendt KW. Obstetric anesthesia and heart disease: practical clinical considerations. Anesthesiology. (2021) 135(1):164–83. 10.1097/ALN.0000000000003833 34046669 PMC8613767

[12] Leffert L Butwick A Carvalho B Arendt K Bates SM Friedman A S. V. T. E. T. Members of the: the society for obstetric anesthesia and perinatology consensus statement on the anesthetic management of pregnant and postpartum women receiving thromboprophylaxis or higher dose anticoagulants. Anesth Analg. (2018) 126(3):928–44. 10.1213/ANE.0000000000002530 29099429

[13] Horlocker TT Vandermeuelen E Kopp SL Gogarten W Leffert LR Benzon HT. Regional anesthesia in the patient receiving antithrombotic or thrombolytic therapy: American society of regional anesthesia and pain medicine evidence-based guidelines (fourth edition). Reg Anesth Pain Med. (2018) 43(3):263–309. 10.1097/AAP.0000000000000763 29561531

[14] Hands ME Johnson MD Saltzman DH Rutherford JD. The cardiac, obstetric, and anesthetic management of pregnancy complicated by acute myocardial infarction. J Clin Anesth. (1990) 2(4):258–68. 10.1016/0952-8180(90)90106-d 2117938

[15] Gil S Atienzar C Filella Y Fernandez M Borras R Miranda A. Anaesthetic management of acute myocardial infarction during labour. Int J Obstet Anesth. (2006) 15(1):71–4. 10.1016/j.ijoa.2005.06.011 16325388

[16] Duarte FP O'Neill P Centeno MJ Ribeiro I Moreira J. Myocardial infarction in the 31st week of pregnancy–case report. Rev Bras Anestesiol. (2011) 61(2):225–7. 10.1016/S0034-7094(11)70027-7 21474030

[17] Pougeoise M Dalmas AF Langlois S Voisin B Dedet B Vaast P Anaesthetic management for caesarean delivery and acute myocardial infarction by spontaneous coronary dissection. Ann Fr Anesth Reanim. (2012) 31(2):162–5. 10.1016/j.annfar.2011.10.025 22154455

[18] Frassanito L Vagnoni S Zanfini BA Catarci S Maggiore S Draisci G. General anesthesia for caesarean delivery in a pregnant woman affected by acute myocardial infarction. Eur Rev Med Pharmacol Sci. (2012) 16(8):1123–6. PMID: 22913165

[19] Mihaljevic S Radivojevic RC Mihaljevic L. Acute coronary syndrome with ST-segment elevation in pregnancy: anesthetic management of delivery. Coll Antropol. (2015) 39(2):447–50. PMID: 26753464

[20] Baris L Hakeem A Moe T Cornette J Taha N Farook F Acute coronary syndrome and ischemic heart disease in pregnancy: data from the EURObservational research programme-European society of cardiology registry of pregnancy and cardiac disease. J Am Heart Assoc. (2020) 9(15):e015490. 10.1161/JAHA.119.015490 32750301 PMC7792249

[21] Lameijer H Kampman MA Oudijk MA Pieper PG. Ischaemic heart disease during pregnancy or post-partum: systematic review and case series. Neth Heart J. (2015) 23(5):249–57. 10.1007/s12471-015-0677-6 25911007 PMC4409591

[22] Larsson C Matsson A Mooe T Soderstrom L Tunon K Nordin P. Cardiovascular complications following cesarean section and vaginal delivery: a national population-based study. J Matern Fetal Neonatal Med. (2021):1–8. 10.1080/14767058.2021.1941851 34275412

[23] Roth A Elkayam U. Acute myocardial infarction associated with pregnancy. J Am Coll Cardiol. (2008) 52(3):171–80. 10.1016/j.jacc.2008.03.049 18617065

[24] Yildirim OI Gunusen I Sargin A Firat V Karaman S. The evaluation of applied anaesthetic techniques for caesarean in parturients with cardiac diseases: retrospective analysis. Turk J Anaesthesiol Reanim. (2014) 42(6):326–31. 10.5152/TJAR.2014.49389 27366446 PMC4894132

[25] Catena E Mele D. Role of intraoperative transesophageal echocardiography in patients undergoing noncardiac surgery. J Cardiovasc Med (Hagerstown). (2008) 9(10):993–1003. 10.2459/JCM.0b013e32830bf655 18799961

[26] Suehiro K Tanaka K Matsuura T Funao T Yamada T Mori T The Vigileo-FloTrac system: arterial waveform analysis for measuring cardiac output and predicting fluid responsiveness: a clinical review. J Cardiothorac Vasc Anesth. (2014) 28(5):1361–74. 10.1053/j.jvca.2014.02.020 25027098

[27] American College of Obstetricians and Gynecologists. Gynecologists’ presidential task force on, D. Heart and B.-O. Committee on practice: ACOG practice bulletin No. 212: pregnancy and heart disease. Obstet Gynecol. (2019) 133(5):e320–56. 10.1097/AOG.0000000000003243 31022123

[28] Fleisher LA Fleischmann KE Auerbach AD Barnason SA Beckman JA Bozkurt B C. American college of and A. American heart: 2014 ACC/AHA guideline on perioperative cardiovascular evaluation and management of patients undergoing noncardiac surgery: a report of the American college of cardiology/American heart association task force on practice guidelines. J Am Coll Cardiol. (2014) 64(22):e77–137. 10.1016/j.jacc.2014.07.944 25091544

[29] Ryu C Choi GJ Jung YH Baek CW Cho CK Kang H. Postoperative analgesic effectiveness of peripheral nerve blocks in cesarean delivery: a systematic review and network meta-analysis. J Pers Med. (2022) 12(4):634. 10.3390/jpm12040634 PMC9033028 35455750

[30] Wang P Chen X Chang Y Wang Y Cui H. Analgesic efficacy of ultrasound-guided transversus abdominis plane block after cesarean delivery: a systematic review and meta-analysis. J Obstet Gynaecol Res. (2021) 47(9):2954–68. 10.1111/jog.14881 34128297

